# Standard Values and Concurrent Validity of a Newly Developed Occlusal Force-Measuring Device among Community-Dwelling Older Adults: The Otassha Study

**DOI:** 10.3390/ijerph19095588

**Published:** 2022-05-04

**Authors:** Masanori Iwasaki, Ichinosuke Maeda, Yota Kokubo, Yoshitomo Tanaka, Tetsuji Ueno, Yuki Ohara, Keiko Motokawa, Misato Hayakawa, Maki Shirobe, Ayako Edahiro, Hisashi Kawai, Yoshinori Fujiwara, Kazushige Ihara, Hunkyung Kim, Yutaka Watanabe, Shuichi Obuchi, Hirohiko Hirano

**Affiliations:** 1 Tokyo Metropolitan Geriatric Medical Center, 35-2 Sakae-cho, Itabashi-ku, Tokyo 173-0015, Japan; yohara@tmig.or.jp (Y.O.); kikiki_1004@yahoo.co.jp (K.M.); erurinrun0424@gmail.com (M.H.); mashirobe@gmail.com (M.S.); aedahiro514@gmail.com (A.E.); hkawai@tmig.or.jp (H.K.); fujiwayo@tmig.or.jp (Y.F.); kimhk@tmig.or.jp (H.K.); ywata@den.hokudai.ac.jp (Y.W.); obuchipc@tmig.or.jp (S.O.); h-hiro@gd5.so-net.ne.jp (H.H.); 2 Sumitomo Riko Co., Ltd., 3-1 Higashi, Komaki-shi, Aichi 485-8550, Japan; ichinosuke.maeda@jp.sumitomoriko.com (I.M.); yota.kokubo@jp.sumitomoriko.com (Y.K.); yoshitomo.tanaka@jp.sumitomoriko.com (Y.T.); tetsuji.ueno@jp.sumitomoriko.com (T.U.); 3 Department of Social Medicine, Hirosaki University Graduate School of Medicine, 5 Zaifu-cho, Hirosaki-shi 036-8562, Japan; ihara@hirosaki-u.ac.jp; 4 Gerontology, Department of Oral Health Science, Faculty of Dental Medicine, Hokkaido University, Kita 13-jo Nishi 7-chome, Kita-ku, Sapporo-shi 060-8586, Japan

**Keywords:** bite force, geriatrics, pressure-mapping sensor, oral health

## Abstract

Recently, an occlusal force-measuring device with a capacitive-type pressure-mapping sensor (OFMD-CPS) was developed. We aimed to establish age- and sex-specific standard values for OFMD-CPS-measured occlusal force (OF) and to assess the concurrent validity of the OFMD against another OF measuring system with a pressure-sensitive sheet (Dental Prescale II). Using data from a population-based study, we calculated the OFMD-CPS-measured OF means and deciles in 5-year age groups for each sex. The OFMD-CPS-measured OF was validated against the Dental Prescale II-measured OF with Spearman correlation coefficients. Furthermore, we calculated the area under the receiver operating characteristic curve (AUC) against the preexisting Dental Prescale II-measured OF cutoff value of 350 N. In total, 596 individuals (236 men and 360 women) with a mean (standard deviation (SD)) age of 73.7 (6.7) years were included in the analyses. The mean (SD) OFMD-CPS-measured OFs were 581.6 (284.6) N in men and 446.9 (209.9) N in women. There was a strong positive correlation (Spearman’s Rho = 0.73) between OFMD-CPS-measured and Dental Prescale II-measured OF. The diagnostic accuracy of the OFMD-CPS-measured OF for the Dental Prescale II-measured OF cutoff value was high (AUC = 0.88). In conclusion, we demonstrated standard values and concurrent validity of OFMD-CPS-measured OF in community-dwelling older adults.

## 1. Introduction

Occlusal force (OF) is an objective measure of oral function. Evidence suggests that OF is a key determinant of masticatory performance [1,2,3]. Oral health is integral to general health and social well-being and refers not only to the number of present teeth but also to functions such as mastication, which is expressed by OF. In Japan, older adults currently retain a larger number of teeth than older adults did previously; the percentage of adults with more than 20 teeth present at 80 years of age exceeded 50% in 2016 [4]. The oral health needs of the older population have changed. In addition to the treatment of tooth loss, dental caries, and periodontal disease, older adults in Japan have a higher need for the assessment and management of their oral functions, including masticatory and swallowing functions.

In response to these changes, in 2018, the Japanese National Health Insurance began covering “oral hypofunction” [5], a seven-component clinical phenotype based on oral health. Since the procedures related to oral hypofunction are now covered by insurance, oral function assessments are accessible to the public in Japan.

OF is one of the diagnostic components of oral hypofunction. To date, one commercially available OF measuring system that consists of a pressure-sensitive sheet (Dental Prescale II, GC Corp., Tokyo, Japan) and dedicated software (Bite Force Analyzer, GC Corp., Tokyo, Japan) has been used to measure OF in the diagnosis of oral hypofunction. Recently, a portable device for OF measurements was developed. This newly developed occlusal force-measuring device has a capacitive-type pressure-mapping sensor and converts mechanical forces into changes in capacitance to calculate OF. The accuracy and repeatability of OF calculations using this device (Occlusal Force-Measuring Device with a Capacitive-type Pressure-mapping Sensor (OFMD-CPS)) were confirmed in a recent study [6].

The current cross-sectional study involving community-dwelling older adults was designed with the aims of (1) determining age- and sex-specific population standard values of OF using the OFMD-CPS and (2) assessing the concurrent validity of the OFMD-CPS using the Dental Prescale II as a reference device. Our secondary aim was to determine a cutoff value for OFMD-CPS-measured OF to diagnose oral hypofunction.

## 2. Materials and Methods

### 2.1. Design, Setting, and Participants

The study population comprised participants from the Otassha Study, an ongoing community-based cohort study involving adults aged ≥ 65 years who were residents of the Itabashi ward, Tokyo, Japan. The protocol of the Otassha Study has been described in detail elsewhere [7]. In addition to a comprehensive assessment of geriatric function, OF measurements and dental examinations were conducted in 2021. These data were used in the current investigation. Individuals who had no occlusal contact between opposing natural or artificial teeth and who had incomplete data were excluded from the analyses.

The Otassha Study was conducted in accordance with the ethical principles of the Declaration of Helsinki and was approved by the Ethics Committee of the Tokyo Metropolitan Institute of Gerontology (reference number: R21-06, date of approval: 27 August 2021). All participants provided written informed consent before participating in the study. The Strengthening the Reporting of Observational Studies in Epidemiology (STROBE) guidelines were followed when reporting the results.

### 2.2. OF Measurements

#### 2.2.1. OF Measurements Using an OFMD-CPS

**Figure 1:**
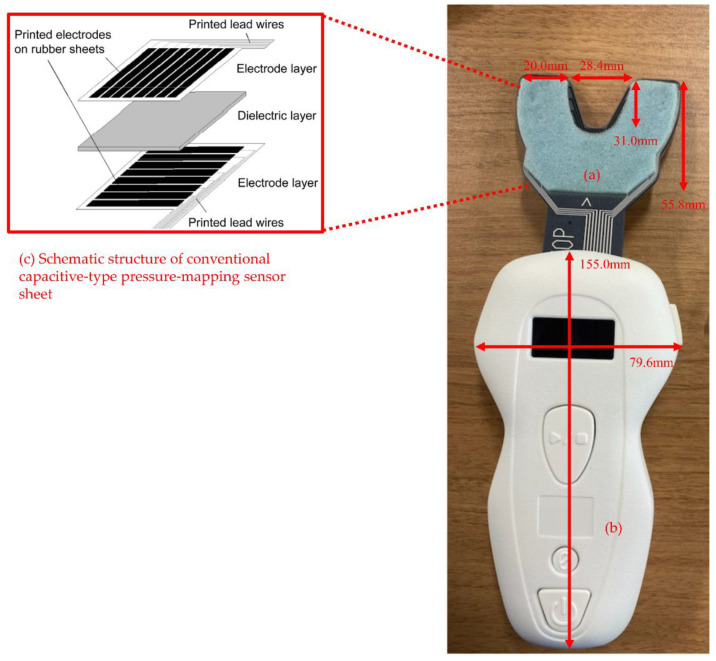
Appearance of occlusal force-measuring device with a capacitive-type pressure-mapping sensor (OFMD-CPS). **Figure 1 legend:** The OFMD-CPS consists of (**a**) a sensor sheet (pressure-sensitive area) and (**b**) a main body with built-in circuit board for the capacitance value measurement. The sensor sheet (**a**) has a three-layer structure, composed of a dielectric layer (urethane sponges) sandwiched between two polymer film sheets (polyethylene terephthalate) as shown in (**c**), and was designed according to the shape of the dental arch. Electrodes were patterned on a polymer film sheet using a screen-printing technique involving special ink. Electrodes were arranged such that the electrodes on the two sides intersected at right angles. One capacitive-type sensor was formed by the intersection of two electrodes. The thickness of the sensor sheet was 1.6 mm, which was reduced to 0.3 mm or less when a bite load was applied.

The bilateral maximal OF was measured using an OFMD-CPS (Figure 1). The participants were asked to perform maximal clenching in the intercuspal position for 3 s, with the sensor sheet of the OFMD-CPS placed between the maxillary and mandibular dental arches. Dentures, if used, were left in for the measurement. The sensor sheet of the OFMD-CPS has a three-layer structure comprising a dielectric layer sandwiched between two electrode layers. Sixty-three pressure-sensitive points are formed by the intersection of 7 electrodes on one side of the electrode layer and 9 on the other side. When a load is applied to the sensor sheet of the OFMD-CPS, the capacitance at each pressure-sensitive point increases as the thickness of the dielectric layer decreases; thus, the capacitance changes owing to the load. The total change in capacitance at each pressure-sensitive point is determined using the vector impedance meter method [8]. Finally, OF is calculated from the total change in capacitance. During clenching, the OF of the participants was continuously calculated and recorded by the OFMD-CPS. The maximum OF value during clenching was used for the analyses. The theory of OF measurements using the OFMD-CPS has previously been described in detail [6].

#### 2.2.2. OF Measurements Using the Dental Prescale II

To measure the bilateral maximal OF, the participants were asked to perform maximal clenching in the intercuspal position for 3 s with the Dental Prescale II placed between the maxillary and mandibular dental arches. Dentures, if used, were left in for the measurement. During clenching, microcapsules contained within the Dental Prescale II collapse, which leads to the intermingling of the color former and the developing agent in the microcapsules. This chemical reaction produces a red color. According to the strength of the pressure applied, different intensities of color are produced. After clenching, the Dental Prescale II was immediately scanned by a dedicated scanner (GT-X830, Seiko Epson Corp., Tokyo, Japan). The OF was calculated by analyzing the scanned image with a Bite Force Analyzer. This software was designed to determine the degree of coloration and the area at each occlusal contact point. To calculate OF, we used the pressure filter function of the Bite Force Analyzer, which automatically excludes areas of red coloration that are likely to have been generated by a force other than occlusal contact [9].

### 2.3. Dental Examination

Six trained dentists and six trained dental hygienists assessed the participants to determine whether each tooth was present, missing and replaced by a dental prosthesis, or missing without replacement. Thereafter, the number of natural teeth was defined as the number of remaining teeth, excluding residual roots. The number of functional teeth was defined as the sum of the number of natural teeth, artificial teeth from dentures, pontics on bridges, and implants [10]. Participants were not examined for other oral conditions, such as dental caries and periodontal disease.

### 2.4. Other Characteristics

Information on the age and sex of the participants was obtained with a self-administered questionnaire.

### 2.5. Statistical Analyses

We described the study population characteristics according to sex. To compare variables, the chi-square test, t test, or Mann–Whitney U test was used, as appropriate. To compare the relative variability, we calculated the coefficients of variation (CVs) for OFMD-CPS-measured and Dental Prescale II-measured OFs.

We tabulated the mean and standard deviation (SD) of OFMD-CPS-measured OF for each 5-year age group (65–69, 70–74, 75–79, 80–84, and ≥85 years) according to sex. We treated OFMD-CPS-measured OF as a continuous variable and conducted a Jonckheere–Terpstra trend test to evaluate linear trends in the mean OFs across age groups for each sex. We further calculated the deciles (10th–90th percentiles) of OF for each 5-year age group for each sex.

Because the numbers of natural teeth and functional teeth did not follow a normal distribution, we used Spearman correlation analyses to assess the relationships among the variables (OF, the numbers of natural teeth and functional teeth, and age).

An OF of 350 N, as measured by the Dental Prescale II and the pressure filter function, was defined as low and used as the cutoff value for oral hypofunction [5]. The predictive validity of OF measured by the OFMD-CPS for this cutoff value was assessed using receiver operating characteristic (ROC) curve analysis. The area under the ROC curve (AUC) was estimated and interpreted as follows: An AUC >0.8 had high predictive power; 0.8 ≥AUC >0.7 had useful predictive power; and an AUC ≤0.7 had low predictive power [11]. The optimal cutoff threshold of OFMD-CPS-measured OF for identifying individuals with low OF was determined using the highest Youden’s Index (sensitivity + specificity − 1).

The presence of <20 natural teeth is an alternative cutoff for a diagnosis of oral hypofunction [5] when the Dental Prescale II-measured OF is not available. We therefore determined another ROC curve for this value (i.e., having <20 natural teeth) and explored the optimal cutoff threshold of OFMD-CPS-measured OF for identifying individuals with fewer teeth.

## 3. Results

### 3.1. Study Population

Overall, 642 individuals participated in the 2021 Otassha Study. Of these, no participants lost occlusal contact, but 46 did not have complete data and were excluded. The remaining 596 adults (360 women (60.4%) and 236 men (39.6%)) were included in the analysis. Their mean (SD) age was 73.7 (6.7) years.

Table 1 presents the characteristics of the participants according to sex. The mean (SD) OFMD-CPS-measured OFs were 581.6 (284.6) N in men and 446.9 (209.9) N in women. The male participants had larger OF values than the female participants. The numbers of natural teeth and functional teeth and age did not significantly differ according to sex. Compared to that of Dental Prescale II-measured OF, the OFMD-CPS-measured OF had a smaller CV.

### 3.2. Descriptive Statistics for OFMD-CPS-Measured OF

In male participants, the mean (SD) OFs for those aged 65–69, 70–74, 75–79, 80–84, and ≥85 years were 581.6 (284.6), 681.4 (262.1), 603.6 (283.3), 551.0 (293.5), 531.2 (265.6), and 294.7 (151.8) N, respectively. In female participants, the corresponding values were 446.9 (209.9), 497.3 (205.1), 433.3 (208.5), 465.6 (210.2), 385.2 (201.8), and 344.8 (196.0) N (Table 2). For both sexes, there was a trend toward a significant decline in OF as age increased.

Table 3 presents the deciles of OF according to age group and sex, displaying the trend toward declining OF with advancing age in both sexes.

### 3.3. Correlations among OF, the Numbers of Natural Teeth and Functional Teeth, and Age

Table 4 shows the Spearman correlations among OF, the numbers of natural teeth and functional teeth, and age. There was a strong positive correlation (Spearman’s Rho = 0.73) between OFMD-CPS-measured OF and Dental Prescale II-measured OF. There were moderate positive correlations (Spearman’s Rho ranged from 0.47 to 0.55) between OFMD-CPS-measured OF and the number of natural teeth, between Dental Prescale II-measured OF and the number of natural teeth, and between the numbers of natural teeth and functional teeth. There was a moderate negative correlation (Spearman’s Rho = −0.31) between the number of natural teeth and age.

### 3.4. Predictive Value of OFMD-CPS-Measured OF

**Figure 2:**
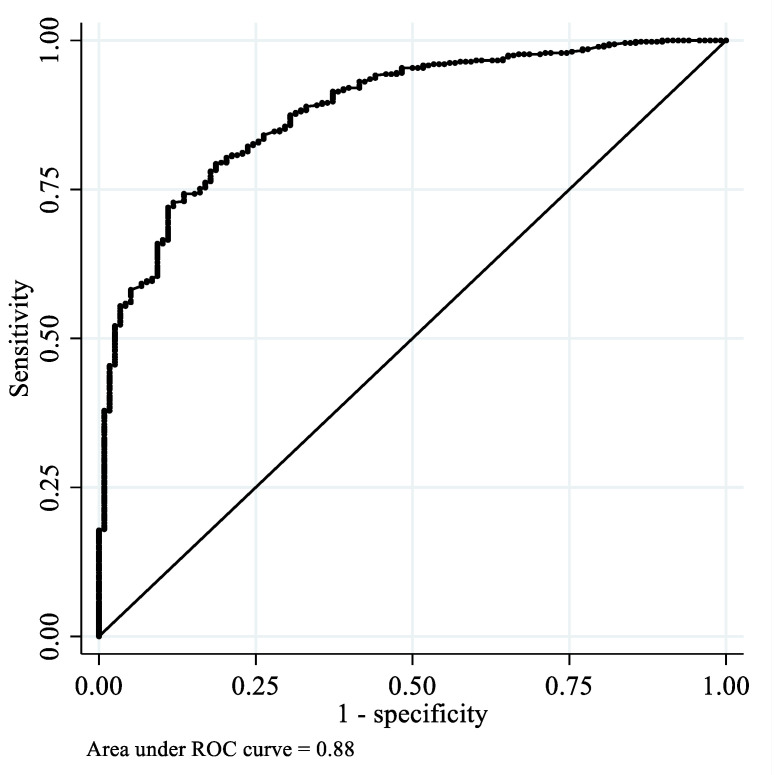
Receiver operating characteristic curves of OFMD-CPS-measured OF among the participants with low OF.

**Figure 3:**
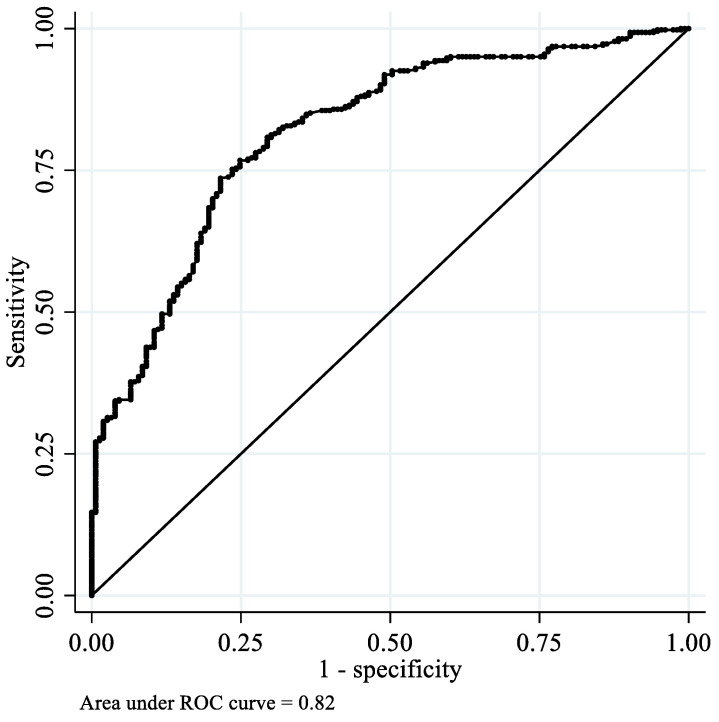
Receiver operating characteristic curves of OFMD-CPS-measured OF among the participants with <20 natural teeth.

The ROC curve of OFMD-CPS-measured OF for low OF is presented in Figure 2. The diagnostic accuracy of OFMD-CPS-measured OF among participants with a low OF was high, with an AUC of 0.88 (95% confidence interval (CI): 0.85–0.91). The cutoff value for OFMD-CPS-measured OF that best differentiated individuals with low OF was 373.5 N, with a sensitivity of 79% and specificity of 81%. Furthermore, the diagnostic accuracy of OFMD-CPS-measured OF among individuals with <20 natural teeth was also high, with an AUC of 0.82 (95% CI: 0.78–0.86; Figure 3). The cutoff score for OFMD-CPS-measured OF was 402 N, with a sensitivity of 75% and specificity of 75%.

## 4. Discussion

In this study, we reported age- and sex-specific standard values for OFMD–CPS-measured OF among community-dwelling older adults. An examination of the associations between OF and other oral functions, such as tongue pressure, factors other than age and sex, or any specific adverse health outcomes, was beyond the scope of our study. OF is associated with masticatory performance regardless of occlusal status [3]. Furthermore, OF is associated with dietary intake, physical and cognitive ability, and mortality among older adults [12,13,14,15,16]. Overall, the measurement of OF is an objective and important method of assessing oral function and is a suitable marker for exploring the connection between oral and systemic health. Therefore, the standard values of OFMD-CPS-measured OF determined in this study can serve as a basis for future studies that use OFMD-CPS-measured OF to assess the risks of nutritional, physical, and cognitive problems. A standard value is also essential for defining low or abnormal values. In this study, we reported OFMD-CPS-measured OF according to sex and different age strata so that clinicians and researchers can reference these values to flexibly define low or abnormal OF values. In addition, the standard values can be utilized to assess the effectiveness of management and interventions for individuals with oral hypofunction. Nonetheless, future longitudinal or intervention studies are needed to determine clinically important differences in OFMD-CPS-measured OF.

Furthermore, this study demonstrated that OFMD-CPS-measured OF had satisfactory validity with regard to Dental Prescale II-measured OF and had a high predictive value for diagnostic components of oral hypofunction (i.e., low OF and few natural teeth) [5]. The results indicate that OFMD-CPS-measured OF can be used to diagnose oral hypofunction.

Recently, the term oral frailty [17,18] has been increasingly adopted and studied. Oral frailty is a new model that focuses on decreases in the multifaceted aspects of function in the oral cavity [18]. Currently, OF is not included in the components of the most frequently used operational definition of oral frailty [18]. However, because OF is an objective measure of oral function and is related to other oral functions, such as masticatory function [19,20], OF could be an important determinant for oral frailty. To assess its importance, further studies on OFMD-CPS-measured OF in relation to other oral functions and/or general health conditions such as physical frailty are needed.

Because the oral health and function of older adults are closely related to their nutritional status and general health [5,17,18,19] and frail older adults have difficulty taking care of their mouths [21], routine oral health assessments are important. In many cases, providing routine assessments has several challenges related to both dental healthcare recipients and providers. Regarding recipients, reduced physical and cognitive conditions and mobility limitations can be a barrier to accessing dental clinics. Regarding providers, complicated techniques for oral function assessments and multiple forms of dental status and general status require substantial time and human resources. This could be particularly applicable to nongerodontological settings. OFMD-CPS measurements are simpler and require less time than other oral function measurements, such as tongue pressure measurements. We should investigate, in the future, whether the OFMD-CPS can make routine oral assessments more accessible.

The accuracy of OFMD-CPS-measured OF has been shown in previous research [6], in which the OF obtained using the OFMD-CPS showed high agreement (R^2^ = 0.99) with the output value of the load cell of the universal testing machine (AG-IS 100 kN, Shimadzu Co., Ltd., Kyoto, Japan). Because the universal testing machine can provide accurate force to the test object [22], the pressure value generated by the universal testing machine constitutes the gold standard.

One difference between the OFMD-CPS and Dental Prescale II is that the OFMD-CPS enables clinicians and researchers to confirm the OF immediately without using other equipment, such as analyzing software or scanners. Additionally, the sensor sheets of the OFMD-CPS are reusable [6], while those of the Dental Prescale II are not. Furthermore, OFMD-CPS-measured OF had a smaller CV than Dental Prescale II-measured OF, indicating that the values obtained from the OFMD-CPS had lower variability and better consistency. On the other hand, several limitations of our study must be considered. First, our study only included individuals living in one specific area of Japan. Sociocultural and economic diversity throughout Japan might affect the OF values. Future studies with broader geographic areas are necessary to investigate potential regional differences. Second, selection bias might have occurred, as participation was voluntary. Individuals with poor physical or mental function tend to avoid participating in community-based surveys [23]. Therefore, our study findings may not be applicable for frailer individuals. Finally, we did not test the interexaminer and intraexaminer agreement for OF measurements using the OFMD-CPS.

In conclusion, the current study determined age- and sex-specific standard values for OF measured using the OFMD-CPS in community-dwelling Japanese adults aged ≥ 65 years. Furthermore, this study demonstrated that OFMD-CPS-measured OF had concurrent validity against Dental Prescale II-measured OF and has the potential to be a user-friendly tool for OF measurements and the diagnosis of oral hypofunction in clinical and epidemiological settings. Exploring the relationship of OFMD-CPS-measured OF with other oral functions, such as tongue pressure, as well as physical functions, such as grip strength, are important next steps to assess the significance of OFMD-CPS-measured OF.

## Figures and Tables

**Table 1:** Characteristics of the study population (n = 596).

	Total	Women	Men	*p* Value
	N = 596	N = 360	N = 236	
OFMD-CPS-measured OF (N) *	500.3 (250.9)	446.9 (209.9)	581.6 (284.6)	<0.01
CV of OFMD-CPS-measured OF	0.50	0.47	0.49	-
Dental Prescale II-measured OF (N) *	814.6 (526.5)	710.9 (439.3)	972.7 (604.5)	<0.01
CV of Dental Prescale II-measured OF	0.65	0.62	0.62	-
Dental Prescale II-measured OF <350 N ^†^	118 (19.8%)	84 (23.3%)	34 (14.4%)	0.01
No. of natural teeth ^‡^	25 (19–28)	25 (20–27)	25 (18–28)	0.65
<20 ^†^ natural teeth	153 (25.7%)	89 (24.7%)	64 (27.1%)	0.51
No. of functional teeth ^‡^	28 (26–28)	28 (26–28)	28 (27–28)	0.31
Age *	73.7 (6.7)	73.4 (6.6)	74.0 (6.9)	0.34

* presented as the mean (SD); † presented as N (%); ‡ presented as the median (IQR); IQR = interquartile range, OF = occlusal force, No. = number, OFMD-CPS = Occlusal Force-Measuring Device with a Capacitive-type Pressure-mapping Sensor, SD = standard deviation.

**Table 2:** Means and standard deviations of occlusal force according to age group and sex.

	Age Group (Years)						
	Total	65–69	70–74	75–79	80–84	≥85	*p* for Trend
Men							
No.	236	72	70	38	36	20	
OF (N) *	581.6 (284.6)	681.4 (262.1)	603.6 (283.3)	551.0 (293.5)	531.2 (265.6)	294.7 (151.8)	<0.01
Women							
No.	360	124	93	56	71	16	
OF (N) *	446.9 (209.9)	497.3 (205.1)	433.3 (208.5)	465.6 (210.2)	385.2 (201.8)	344.8 (196.0)	<0.01

SD = standard deviation, OF = occlusal force, No. = number; * presented as the mean (SD).

**Table 3:** Deciles of occlusal force by age group and sex.

		Age Group (Years)					
	Decile	Total	65–69	70–74	75–79	80–84	≥85
Men (No. = 236)							
	90th	944	954	944	945	811	526
	80th	855	906	873	795	761	440
	70th	763	855	787	752	684	406
	60th	657	763	687	670	551	302
	50th (Median)	568	668	587	530	497	244
	40th	484	626	520	426	461	230
	30th	413	550	451	339	327	174
	20th	310	428	372	254	287	149
	10th	215	347	208	197	232	135
Women (No. = 360)							
	90th	743	751	756	770	667	640
	80th	608	660	585	652	547	510
	70th	549	573	500	579	496	491
	60th	483	541	472	512	455	401
	50th (Median)	435	478	424	430	399	316
	40th	390	442	371	401	312	226
	30th	329	392	329	350	246	212
	20th	262	325	278	301	158	188
	10th	158	247	151	186	145	118

No. = number.

**Table 4:** Correlation between OF, the numbers of natural teeth and functional teeth, and age.

Variables		1	2	3	4
1	OFMD-CPS-measured OF				
2	Dental Prescale II-measured OF	0.73 *			
3	No. of natural teeth	0.53 *	0.55 *		
4	No. of functional teeth	0.16 *	0.18 *	0.47 *	
5	Age	−0.25 *	−0.14 *	−0.31 *	−0.07

Spearman’s Rho is presented. * *p* < 0.05; OF = occlusal force, No. = number, OFMD-CPS = Occlusal Force-Measuring Device with a Capacitive-type Pressure-mapping Sensor.

## Data Availability

The data presented in this study are available upon request from the corresponding author. The data are not publicly available due to ethical and legal restrictions imposed by the Ethics Committee of the Tokyo Metropolitan Institute of Gerontology.

## References

[1] Hatch J.P., Shinkai R.S., Sakai S., Rugh J.D., Paunovich E.D. (2001). Determinants of masticatory performance in dentate adults. Arch. Oral Biol..

[2] Ikebe K., Matsuda K., Kagawa R., Enoki K., Okada T., Yoshida M., Maeda Y. (2012). Masticatory performance in older subjects with varying degrees of tooth loss. J. Dent..

[3] Kosaka T., Ono T., Kida M., Kikui M., Yamamoto M., Yasui S., Nokubi T., Maeda Y., Kokubo Y., Watanabe M. (2016). A multifactorial model of masticatory performance: The Suita study. J. Oral Rehabil..

[4] The Ministry of Health, Labour, and Welfare Survey of Dental Diseases, 2016 (In Japanese). https://www.mhlw.go.jp/toukei/list/dl/62-28-02.pdf..

[5] Minakuchi S., Tsuga K., Ikebe K., Ueda T., Tamura F., Nagao K., Furuya J., Matsuo K., Yamamoto K., Kanazawa M. (2018). Oral hypofunction in the older population: Position paper of the Japanese Society of Gerodontology in 2016. Gerodontology.

[6] Iwasaki M., Maeda I., Kokubo Y., Tanaka Y., Ueno T., Takahashi W., Watanabe Y., Hirano H. (2022). Capacitive-type pressure-mapping sensor for measuring bite force. Int. J. Environ. Res. Public Health.

[7] Fujiwara Y., Suzuki H., Kawai H., Hirano H., Yoshida H., Kojima M., Ihara K., Obuchi S. (2013). Physical and sociopsychological characteristics of older community residents with mild cognitive impairment as assessed by the Japanese version of the Montreal Cognitive Assessment. J. Geriatr. Psychiatry Neurol..

[8] Agilent Technologies Agilent Impedance Measurement Handbook. https://indico.cern.ch/event/216963/sessions/35851/attachments/347577/484629/impedancemeasurementhandbook_2.pdf.

[9] Horibe Y., Matsuo K., Ikebe K., Minakuchi S., Sato Y., Sakurai K., Ueda T. (2021). Relationship between two pressure-sensitive films for testing reduced occlusal force in diagnostic criteria for oral hypofunction. Gerodontology.

[10] Murakami M., Iijima K., Watanabe Y., Tanaka T., Iwasa Y., Edahiro A., Ohara Y., Motokawa K., Shirobe M., Hirano H. (2020). Development of a simple method to measure masseter muscle mass. Gerodontology.

[11] Carra M.C., Gueguen A., Thomas F., Pannier B., Caligiuri G., Steg P.G., Zins M., Bouchard P. (2018). Self-report assessment of severe periodontitis: Periodontal screening score development. J. Clin. Periodontol..

[12] Ikebe K., Gondo Y., Kamide K., Masui Y., Ishizaki T., Arai Y., Inagaki H., Nakagawa T., Kabayama M., Ryuno H. (2018). Occlusal force is correlated with cognitive function directly as well as indirectly via food intake in community-dwelling older Japanese: From the SONIC study. PLoS ONE.

[13] Inomata C., Ikebe K., Kagawa R., Okubo H., Sasaki S., Okada T., Takeshita H., Tada S., Matsuda K., Kurushima Y. (2014). Significance of occlusal force for dietary fibre and vitamin intakes in independently living 70-year-old Japanese: From SONIC Study. J. Dent..

[14] Ohi T., Komiyama T., Miyoshi Y., Murakami T., Tsuboi A., Tomata Y., Tsuji I., Watanabe M., Hattori Y. (2018). Maximum occlusal force and incident functional disability in older adults: The Tsurugaya project. JDR Clin. Trans. Res..

[15] Okada T., Ikebe K., Kagawa R., Inomata C., Takeshita H., Gondo Y., Ishioka Y., Okubo H., Kamide K., Masui Y. (2015). Lower protein intake mediates association between lower occlusal force and slower walking speed: From the septuagenarians, octogenarians, nonagenarians investigation with centenarians study. J. Am. Geriatr. Soc..

[16] Iwasaki M., Yoshihara A., Sato N., Sato M., Taylor G.W., Ansai T., Ono T., Miyazaki H. (2016). Maximum bite force at age 70 years predicts all-cause mortality during the following 13 years in Japanese men. J. Oral Rehabil..

[17] Dibello V., Zupo R., Sardone R., Lozupone M., Castellana F., Dibello A., Daniele A., De Pergola G., Bortone I., Lampignano L. (2021). Oral frailty and its determinants in older age: A systematic review. Lancet Healthy Longev..

[18] Tanaka T., Takahashi K., Hirano H., Kikutani T., Watanabe Y., Ohara Y., Furuya H., Tetsuo T., Akishita M., Iijima K. (2018). Oral Frailty as a Risk Factor for Physical Frailty and Mortality in Community-Dwelling Elderly. J. Gerontol. A Biol. Sci. Med. Sci..

[19] Iwasaki M., Hirano H., Ohara Y., Motokawa K. (2021). The association of oral function with dietary intake and nutritional status among older adults: Latest evidence from epidemiological studies. Jpn. Dent. Sci. Rev..

[20] Iwasaki M., Motokawa K., Watanabe Y., Shirobe M., Ohara Y., Edahiro A., Kawai H., Fujiwara Y., Kim H., Ihara K. (2021). Oral hypofunction and malnutrition among community—dwelling older adults: Evidence from the Otassha study. Gerodontology.

[21] Petersen P.E., Kandelman D., Arpin S., Ogawa H. (2010). Global oral health of older people--call for public health action. Community Dent Health.

[22] Shimadzu Cooperation Shimadzu Universal Testers. https://www.quark-gulf.com/quark/public/storage/Products/gynGTetHuFjf0qnYm1f2BDZekbESroVJdtgRWX8n.pdf..

[23] Kan M., Yoshida H., Fujiwara Y., Watanabe N., Tsuchiya Y., Shinkai S. (2006). Longitudinal analysis of factors associated with participation in community-based mass screening for the frail elderly in need of care. Jpn. J. Public Health.

